# Unraveling Brain Synchronisation Dynamics by Explainable Neural Networks using EEG Signals: Application to Dyslexia Diagnosis

**DOI:** 10.1007/s12539-024-00634-x

**Published:** 2024-07-02

**Authors:** Nicolás J. Gallego-Molina, Andrés Ortiz, Juan E. Arco, Francisco J. Martinez-Murcia, Wai Lok Woo

**Affiliations:** 1 Communications Engineering Department, University of Málaga, 29004 Málaga, Spain; 2 Department of Signal Theory, Networking and Communications, University of Granada, 18010 Granada, Spain; 3 Department of Computer and Information Sciences, Northumbria University, Newcastle Upon Tyne, NE1 8ST UK; 4 Andalusian Research Institute in Data, Science and Computational Intelligence, 18010 Granada, Spain; 5 Research Centre for Information and Communication Technologies (CITIC-UGR), University of Granada, 18010 Granada, Spain

**Keywords:** Cross-frequency coupling, Brain synchronisation dynamics, Explainability, Dyslexia

## Abstract

**Abstract:**

The electrical activity of the neural processes involved in cognitive functions is captured in EEG signals, allowing the exploration of the integration and coordination of neuronal oscillations across multiple spatiotemporal scales. We have proposed a novel approach that combines the transformation of EEG signal into image sequences, considering cross-frequency phase synchronisation (CFS) dynamics involved in low-level auditory processing, with the development of a two-stage deep learning model for the detection of developmental dyslexia (DD). This deep learning model exploits spatial and temporal information preserved in the image sequences to find discriminative patterns of phase synchronisation over time achieving a balanced accuracy of up to 83%. This result supports the existence of differential brain synchronisation dynamics between typical and dyslexic seven-year-old readers. Furthermore, we have obtained interpretable representations using a novel feature mask to link the most relevant regions during classification with the cognitive processes attributed to normal reading and those corresponding to compensatory mechanisms found in dyslexia.

**Graphical Abstract:**

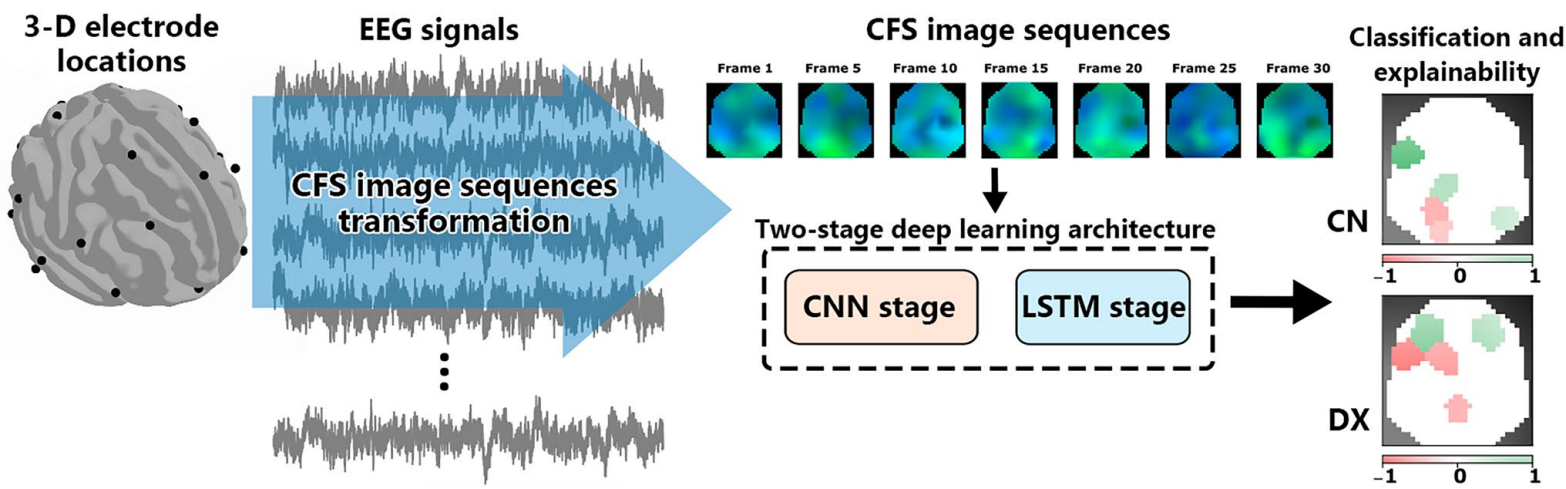

## Introduction

Neuronal oscillations are involved in human cognition and their study helps us understand the brain processes that originate in cognitive functions. In the case of language and speech processing, neuronal oscillations in the auditory cortex provide biophysical constraints and act as a crucial component for the parsing and decoding of connected speech [1]. During the development of language skills, the brain has to learn to extract and discriminate meaningful speech units such as phonemes, syllables, and prosodic stress patterns. By performing temporal sampling of the speech signal at multiple time-scales, the brain can simultaneously differentiate these phonological elements [1, 2]. Although this learning process is rather complex, the brain of typically developing individuals automatically recognises variations in the fundamental units of speech even in a changing context [3]. On the other hand, we encounter developmental disorders such as developmental dyslexia (DD) that hinder this ability.

DD is a specific difficulty in the learning process of reading and spelling not related to mental age or inadequate schooling. This learning disability affects 5%–12% of the world’s population [4] and has a significant impact on school failure and self-esteem of affected children [5]. A phonological deficit can be found in all languages before this impaired learning process starts. Individuals with dyslexia have difficulties in discriminating meaningful speech units. The underlying cause of these difficulties is reflected in alterations in the mechanisms of the neural oscillations that encode input information at multiple frequencies. This process fits within the temporal sampling framework of the speech signal [6] that states that a typical developing brain tracks the temporal structure of the speech using neural oscillations on multiple time scales: delta (0.5–4 Hz) for prosodic rhythms, theta (4–8 Hz) for syllabic rhythms and gamma oscillations (30–60 Hz) for phonemic information [1].

The integration of information across multiple spatiotemporal scales can be studied through a complex regulatory structure, the CFC phenomenon [7]. This mechanism is based on the coupling between different brain rhythms that coordinate neural dynamics involved in healthy and pathological brain functions. CFC analyses have been used to explore and understand the impaired brain processes involved in DD using different brain data techniques [8, 9]. Specifically, electroencephalography (EEG) has been widely used to explore CFC [10–12]. This non-invasive and cost-effective technique allows information to be extracted from the neural oscillations involved in brain processes. As a result, functional brain activity can be inferred from EEG signals, and biomarkers are sought that reveal useful insights to better comprehend the neural basis of DD. Nevertheless, it is not straightforward to identify differential functional brain patterns in EEG signals, and it also differs with the specific condition and the particular stimuli applied.

One way to assess the presence of these patterns in the extracted information is using machine learning methods. In particular, deep learning models support deployment on a broad range of medical data. For instance, in [13] peripheral blood smear images were employed to train an improved ResNet50 convolutional neural network (CNN) model for acute lymphoblastic leukaemia diagnosis, in [14] a feature fusion approach applied to magnetic resonance imaging (MRI) was proposed for brain tumour detection, [15] performed an analysis of retinal fundus images with artificial bee colony algorithm and active deep learning for the detection of diabetic retinopathy, and [16] applied a novel feature selection technique to COVID-19 data. Furthermore, by using non–invasive neuroimage techniques, deep learning algorithms can improve the detection of impaired cognitive processes in brain disorders. Deep learning techniques have been employed together with EEG signals to detect epileptic seizures [17, 18], Parkinson’s disease [19], Alzheimer’s disease [20], mild cognitive impairment [21], major depressive disorder [22], and autism spectrum disorder [23]. The usage of machine learning for DD is increasing, allowing the search of characteristic patterns in EEG signals arising from the defective speech encoding in DD. Table 1 presents a comparison of works using EEG and machine learning for dyslexia diagnosis. Among all these uses of deep learning, a challenging part is defining the inputs [24]. The major challenge here is not only to achieve a classification of the extracted information in the EEG data, but to uncover the most meaningful information in order to better grasp the distinctions between healthy and pathological brain functions.

**Table 1:** Comparison of works relying on EEG signals and machine learning for dyslexia diagnosis

Refs	*N*	Stimulus	Input features	Machine learning method	Balanced accuracy
[25]	48	Auditory	Autoencoder residuals	Autoencoder and SVC	93%
[26]	40	Rest	EEG spectral features	ANN	97.5%
[27]	32	Typing and writing	EEG spectral features	SVM	77.5%
[28]	48	Auditory	Z–layer features	Autoencoder and SVC	69.7%
[29]	50	Auditory	EEG spectral features	SVM	78.6%
[30]	26	Auditory and visual	EEG spectral features	Random forest classifier	96.2%

In our work, we propose an approach to learn high-level representations from EEG data that allows the identification of discriminative descriptors of DD. Given the importance of CFC in the coordination of neural dynamics involved in brain functions, we have built a deep learning model around an exploratory analysis of the EEG signals using CFS to determine if altered neural oscillation mechanisms that encode speech signals are present in the phonological deficit in DD. First, to show up this deficit, we have synthesised noninteractive auditory stimuli at rates linked to the fundamental phonological units of the Spanish language by means of amplitude-modulated white noise. The EEG signals acquired in this experiment are then used to perform an exploratory analysis of the CFS between intra-electrode brain rhythms. The main novelty of this contribution is based on the use of this CFS analysis to perform a transformation of EEG signals into image sequences that arise from brain processes developed during auditory processing. In addition, the transformation ensures the preservation of temporal information from the EEG signals and, more importantly, the spatial information that is usually missed in EEG studies.

This transformation into CFS image sequences allows the development of a two-stage deep learning architecture that exploits the spatial and temporal information embedded in them. Simultaneously, they involve valuable information on phase synchronisation over time that is used by the proposed model to find discriminative patterns corresponding to the altered neural processes in low-level language processing in DD. Finally, we have employed and adapted the local interpretable model-agnostic explanations (LIME) interpretability method to the structure of the CFS image sequences using a novel feature mask to improve the understanding of the neural basis of DD by identifying the most relevant regions during classification. The remaining parts of the paper are structured as follows. First, in Sect. 2 we describe the database and methodology. In Sect. 3, we present the main results, and in Sect. 4, these are discussed and interpreted. Lastly, main conclusions are given in Sect. 5

## Materials and Methods

### Dataset and Preprocessing

This work employs data collected by the LEEDUCA research group at the University of Málaga (Spain) [25]. The included participants were selected from a cohort of a longitudinal study of over 1400 children aged 4–8 years. A subcohort of 33 children with no obvious impairment and 15 children with a formal diagnosis of dyslexia was selected through the termly application of a full set of cognitive and linguistic tests. All selected participants had matching ages and a school-level socioeconomic index measured on a scale of 1–10. Children in the EEG subcohort were exposed to amplitude-modulated white noise at frequencies corresponding to those estimated for three core speech units in the Spanish language: syllables (4.8 Hz), intra-syllabic rhythms (16 Hz) and phonemes (40 Hz). Each participant was given 15-minute periods with the presentation of stimuli in an increasing and decreasing sequence for 2.5 min each (4.8 – 16 – 40 – 40 – 16 – 4.8 Hz). EEG data have been recorded using a sampling frequency of 500 Hz with the Brainvision actiCHamp Plus with actiCAP (Brain Products GmbH, Germany) with 32 electrodes following a 10–20 configuration optimised for auditory processing. After acquisition, Independent component analysis (ICA) blind source separation was used to remove artefacts caused by eye blinks. Each channel signal was normalised to zero mean and unit variance and referenced to the Cz electrode. A baseline correction was also carried out. The data utilized in this investigation were acquired through experimental procedures conducted with the explicit consent of the legal guardians of each child, overseen by their presence. Approval for the study was obtained from the Medical Ethical Committee of the University of Málaga (reference: CEUMA 16-2020-H), with strict adherence to the dispositions outlined in the World Medical Association Declaration of Helsinki. Furthermore, the Education Department of the Regional Government of Andalusia (Spain) provided formal endorsement, granting authorization to our researchers for conducting the study within several public school settings.

### Cross–Frequency Phase Synchronisation

In neural oscillations, we encounter the presence of distinct brain rhythms corresponding to band-specific activity and provide temporal windows for different cognitive processes. Communication and interaction between multiple processes taking place in different frequency bands are governed by a complex regulatory structure, the CFC. We explore the phase–phase CFC approach, in particular, CFS at the same location. This type of CFC plays a role as a potential mechanism to regulate communication between neural activity that occurs at different rates [7]. The method proposed here to analyse CFS is a variation of the intersite phase clustering (ISPC) [31] and relies on the distribution of phase-angle differences between different EEG rhythm in the same electrode. As in other phase-based coupling analyses, we consider that when a functional coupling exists between brain rhythms the timing of their oscillatory processes becomes synchronised.

**Fig. 1:**
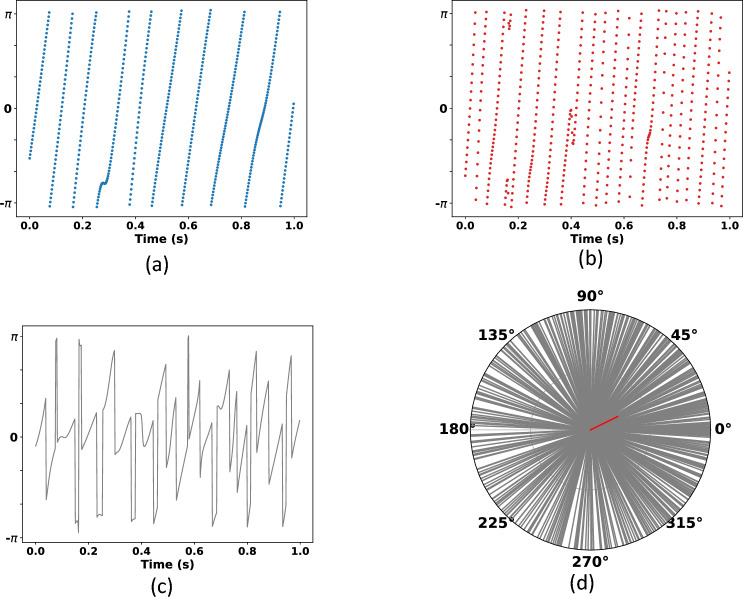
Example of CFS computation. $\phi _{A}(t)$ is the phase angle corresponding to the frequency band *A* and $\phi _{B}(t)$ to *B*. The CFS measure (denoted by *S*_CF_) is the length of the red vector (average of phase differences) in the distribution of phase angle differences (d)

The phase angles of the EEG signals provide information about the timing of frequency-band-specific activity and can be represented as vectors with unit length on a polar plane. In Fig. 1a and b we have two examples of the phase angles for two frequency bands of a segment from an EEG signal. Instead of representing these two phase angle populations in a polar space, we take the phase angle differences (Fig. 1c). Thus, representing the corresponding unit vectors constitutes the distribution of phase angle differences (Fig. 1d). In our case, we measured the clustering in the polar space of these phase angle differences by computing the length of the average of phase angle differences between frequency bands in the same electrode over time.

1 $$\begin{aligned} {S_{\text{CF}}} = \left| \frac{1}{n}\sum ^{n}_{t=1}{\text{e}^{\text{i}(\phi _{A}(t)-\phi _{B}(t))}}\right| \end{aligned}$$

where for the frequency bands *A* and *B*, the phase angles are $\phi _{A}(t)$ and $\phi _{B}(t)$, and *n* indicates the time points employed.

### CFS Image Sequences

The method defined above paves the way for creating a set of image sequences that contain CFS information. First, the preprocessed EEG signals are bandpass filtered to extract the EEG frequency bands. Filter selection is essential to avoid phase distortion, as we are interested in the phase of the EEG signal. In this case, finite input response (FIR) filters offer significant advantages over infinite input response (IIR) filters [32]. IIR filters introduce a nonlinear phase response in the pass band, time shifting the frequency components in the band-pass signal by a variable amount of time, resulting in a distortion of the phase relationships in the filtered signal. FIR filters introduce phase lag over the pass-band as well. However, this delay is constant and can be corrected afterwards by shifting the signal by a constant number of samples. We used FIR filters with a windowed time-domain approach with Hamming window to extract the five EEG frequency bands for each EEG channel. The cut-off frequencies and filter length for each band are 0.5–4 Hz and 3301 samples for Delta, 4–8 Hz and 825 samples for Theta, 8–12 Hz and 825 samples for Alpha, 12–30 Hz and 551 samples for Beta, and 30–80 Hz and 221 samples for Gamma.

Then, to explore the CFS we need the time-varying phase values of the bandpass signals. This is known as the instantaneous phase, $\phi (t)$, and it is computed from the analytic signal, *z*(*t*). This is a complex-valued time series without negative frequency components and is formulated for any given signal *x*(*t*) as

2 $$\begin{aligned} z(t) = x(t) + {\text{j}}H[x(t)] \end{aligned}$$

where *H*[*x*(*t*)] corresponds to the Hilbert transform (HT) of the signal *x*(*t*)

3 $$\begin{aligned} H[x(t)] = \frac{1}{\pi }\int ^{+\infty }_{-\infty }{\frac{x(t)}{t-\tau }d\tau } \end{aligned}$$

The analytic signal *z*(*t*) allows us to estimate the instantaneous, unwrapped phase $\phi (t)$ as

4 $$\begin{aligned} \phi (t) = \angle {z(t)} = \tan ^{-1}\frac{\Im {(z(t))}}{\Re {(z(t))}} \end{aligned}$$

**Fig. 2:**
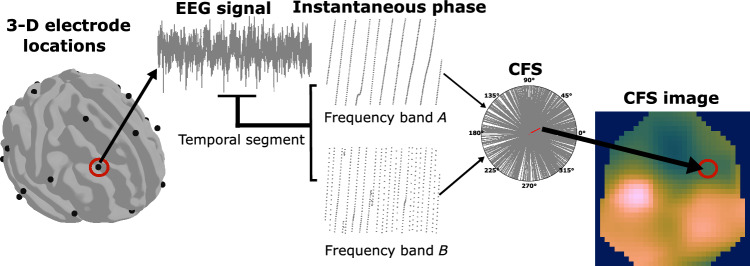
Schema of the proposed methodology. The electrode locations are projected to a 2-D surface where the CFS values are represented. Thus, creating CFS images for each temporal segment of the EEG signal. These CFS images are composed of three layers each one with the information of the CFS between two pairs of frequency bands. Here, we show the creation of one of these layers with the measures of the CFS between the instantaneous phases of frequency band *A* and *B*, namely Delta, Theta, Alpha, Beta, and Gamma

The CFS sequences require the segmentation of the $\phi (t)$ obtained. Using a non-overlapping rectangular window of 5 s long we compute 30 segments from the original instantaneous phase in each EEG frequency band and channel. Then, we explore the CFS measuring the phase syncronisation between the five EEG frequency bands in each EEG channel and in every segment. Thus, in a segment, the CFS is calculated between pairs of frequency bands in the EEG signal. Having done this for every channel, we have a set of 31 CFS values for each pair of frequency bands (Fig. 2). These sets of CFS values represent the phase synchronisation in every EEG channel between a frequency band *A* and *B* in a temporal segment.

Instead of representing these features as a vector, we obtain an image sequence preserving the spatial information of EEG channel location and including insights into the temporal development of CFS involved in cognitive processes. This process of transforming CFS values from a couple of frequency bands in a temporal segment onto a 2-D image is as follows: First, we have to project the 3-D location of the EEG electrodes onto a plane. The EEG electrode locations, distributed over the scalp, can be approximated by a sphere. Thus, we can apply spherical projections to transform 3-D electrode locations into 2-D projected locations as proposed by [33]. The projection method selected is the azimuthal equidistant projection [34], which is widely used in map projection. It has the characteristics that the distances are preserved and all directions are correct when measured from the centre of the projection to any other point. However, relative distances are not exactly preserved. The centre point here corresponds to the Cz electrode. A given point is projected considering its true azimuth, $\theta$, and at a distance from the centre of the map proportional to the distance on the sphere, $\rho = Rc$, where *c* is the angular distance from the centre and *R* is the radius of the sphere. Thus, the Cartesian coordinates of the point on the plane specified by ($\theta$,$\rho$) are

5 $$\begin{aligned} x= & {} \rho \sin {\theta } \end{aligned}$$

6 $$\begin{aligned} y= & {} -\rho \cos {\theta } \end{aligned}$$

Once we have the projected coordinates of the locations of the electrodes on a 2-D surface, we can represent the CFS measurements for each EEG channel. Therefore, we have 31 CFS values scattered in this 2-D surface. Then, we estimate the values in between the EEG channel coordinates by applying a Clough-Tocher interpolation scheme [35] over a $32\times 32$ mesh. Thus, a CFS image is generated with $32\times 32$ pixels. This process is repeated for every 5-second segment that corresponds to a frame of the final CFS sequence. Then, we proceed in the same way for the rest of combination of frequency bands. We finally constructed three-channel CFS images by merging 2-D images produced for three selected pairs of frequency bands in each case. The image resulting from the CFS measured in each frequency band pair is taken as a layer. For example, we have channel 1 for Delta-Theta CFS, channel 2 for Alpha-Beta CFS, and channel 3 for Beta–Gamma CFS. Creating an RGB CFS image for each temporal segment we compose a sequence of images that represent the development of the phase synchronisation together with the combined information of the different frequency bands used with the spatial information of the EEG channel locations.

### Classification and Interpretability

Once the above process has been completed, each subject has an image sequence consisting of 30 CFS images of $32\times 32$ pixels. Each image has phase synchronisation information between three pairs of frequency bands at each electrode, and we can use the information from the CFS image sequences to extract spatial and phase synchronisation representations from each frame and temporal patterns from the sequence. Therefore, it is a key point to develop a two-stage deep learning architecture that translates all this information into high-level representations.

#### Neural Network

After the generation of the CFS image sequences, it is essential to develop a framework that allows the analysis of those images. One of the most relevant aspects of the transformation into images is that it is possible to apply a number of methodologies developed for this data modality. Nevertheless, it is relevant to acknowledge that the temporal information provided by the EEG signals is not discarded during the transformation process to images. To take advantage of the spatial and temporal information embedded in the images, we designed a two–stage architecture built on deep learning. This design draws on the architecture proposed in [33] which we have adapted to our specific research objectives, based on previous experience, by exploring different configurations and parameters to optimise performance and generalisation ability through cross–validation. This process has been implemented mainly with the Python libraries Scikit-learn [36], Pytorch [37] and Captum [38]. The generated images were first entered into a CNN that acts as a feature extractor. This means that it performs a dimensionality reduction from the original input space (image of $N \times M$) to a new one. This alternative is commonly used in the processing of images given the robustness that these approaches have in the extraction of representations of input patterns [39, 40]. The convolutional layer is primary element of a CNN. This operator employs a tensor $\varvec{V}_{i-1}$ which contains the activation map from the preceding layer $i-1$. Therefore, a set of *N* filters $\varvec{W}_i$ are implied in the learning process of the target layer (*i*) with a bias factor $\varvec{b}_i$, as defined below:

7 $$\begin{aligned} \varvec{V}_i = f_{\text{a}}(\varvec{W}_i*\varvec{V}_{i-1}+\varvec{b}_i) \end{aligned}$$

where $f_{\text{a}}(*)$ is the activation function. Considering a two-dimensional tensor ($\varvec{V}_{i-1}$) of size $H \times W \times C$ (height, width, and number of channels, respectively), $\varvec{W}_i$ is of size $P \times Q \times S \times K$ where *K* is the count of filters. The *k*th convolution term for the *k*th filter is given as follows:

8 $$\begin{aligned} \varvec{W}_{ik} * \varvec{V}_{i-1} = \sum _{u=0}^{P-1} \sum _{v=0}^{Q-1} [\varvec{W}_{ik}(P-u, Q-v) \cdot \varvec{V}_{i-1}(x+u,y+v)] \end{aligned}$$

**Fig. 3:**
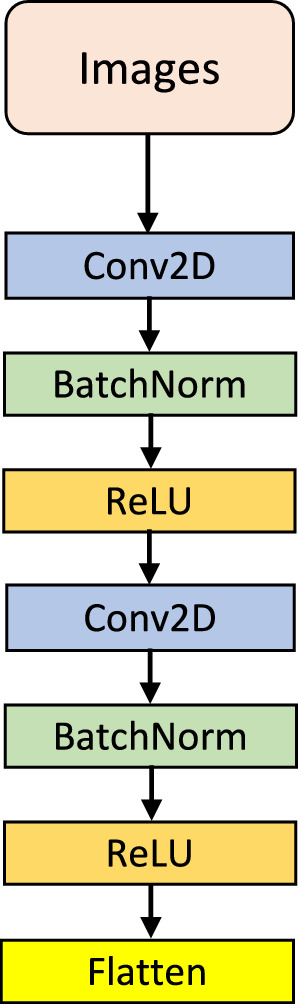
Scheme of the CNN employed for feature extraction. It comprises two convolutional layers, each of them followed by a batch normalisation and ReLU activation to accelerate network convergence. Finally, a flattening is applied to adapt the dimensionality of the output of the network to the next stage of the framework

In layer *i*, the activation of the filters is saved and passed on to the subsequent layer, $i+1$, after the convolution has been performed. Figure 3 shows the architecture of the CNN used. After each convolutional layer, batch normalisation is used to accelerate convergence [41]. Then, a rectified linear activation (ReLU) function is employed to overcome the vanishing gradient problem [42]. At the end of the stack of convolutional layers, a flattening is applied to adapt the dimensionality of the output of the network to the next stage of the framework.

As mentioned above, CNN addresses the spatial location of the information. However, EEG signals are always used for the temporal resolution that they offer. The transformation from EEG data to images proposed in this work is extremely relevant because it preserves the temporal information that the original signals have. To analyse this temporal nature, we employed a long-short term memory (LSTM) model that was fed with the output of the CNN architecture described above. The LSTM, which shows high robustness against the problem of vanishing and exploding gradients [43, 44], is a variant of recurrent neural network (RNN). The idea behind RNN is that sequences of inputs are not independent as in traditional neural networks. Specifically, an RNN maintains some kind of state, which means that its output could be used as part of the next input, leading to a sequential propagation of the information. In fact, there are situations in which the inference about the current situation depends on what happened previously. This important aspect is covered in RNN by the concept of hidden state. For each element of a sequence of information, there is a corresponding hidden state, $h_t$, which contains information about previous arbitrary points of the sequence.

The storage or removal of the information in an LSTM cell is regulated by the inclusion of three gates: the forget, the input, and the output gates. First, the forget gate chooses whether the information has to be skipped according to the entry value derived from the previous state and the current input. Thus, the output of a forget gate at a time *t* is computed as follows:

9 $$\begin{aligned} {\varvec{f}}_{t} = \sigma ({\varvec{W}}_{f}[{\varvec{h}}_{t-1},{\varvec{x}}_t]+{\varvec{b}_i} \end{aligned}$$

where $\sigma$ represents the sigmoid function, ${\varvec{W}}_f$ refers to the weight, $\varvec{h}_{t-1}$ is the hidden state at time $t-1$, $\varvec{x}_{t}$ denotes the input at time *t* and $\varvec{b}_i$ represents the bias value.

Next, the input gate manages the storage of new information in the cell state at a specific time *t*. This process is divided into two steps: first, a sigmoid layer is used to decide the values to be updated. Second, a tanh layer decides the cell, $\tilde{\varvec{C}}_{t}$, that is going to be added to the cell state. These operations can be mathematically expressed as follows:

10 $$\begin{aligned} \varvec{i}_{t}&= \sigma (\varvec{W}_{i}[\varvec{h}_{t-1}, \varvec{x}_t]+ \varvec{b}_i) \end{aligned}$$

11 $$\begin{aligned} \tilde{\varvec{C}}_{t}&= \text{tanh}(\varvec{W}_{\varvec{C}}[\varvec{h}_{t-1}, \varvec{x}_t]+ \varvec{b}_{\varvec{C}}) \end{aligned}$$

Finally, the output of the LSTM cell is decided by the output gate. At time *t*, the updated cell state $\varvec{C}_t$ and the hidden state $\varvec{h}_t$ are computed as follows:

12 $$\begin{aligned} \varvec{C}_t&= \varvec{f}_t \cdot \varvec{C}_{t-1} + \varvec{i}_t \cdot \tilde{\varvec{C}}_t \end{aligned}$$

13 $$\begin{aligned} \varvec{o}_t&= \sigma (\varvec{W}_{\varvec{o}}[\varvec{h}_{t-1}, \varvec{x}_t]+ \varvec{b}_{\varvec{o}}) \end{aligned}$$

14 $$\begin{aligned} \varvec{h}_t&= \varvec{o}_t \cdot \text{tanh}(\varvec{C}_t) \end{aligned}$$

**Fig. 4:**
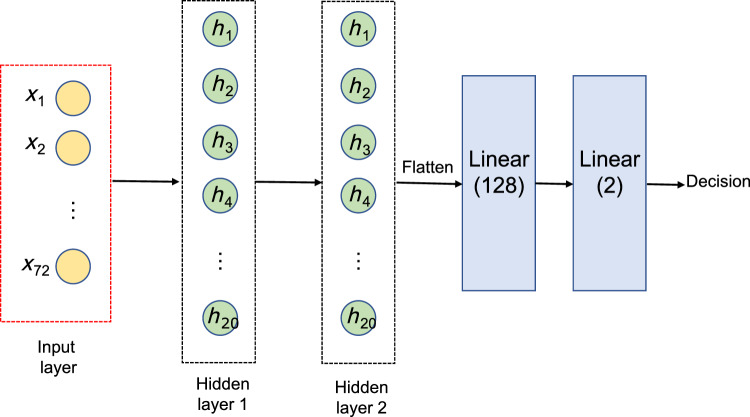
Diagram of the LSTM network. It consists of an input layer of the same size as the output of the CNN network and two hidden layers with 20 LSTM units. Once the output is flattened, it is entered into two fully-connected layers, the last one with two output neurons representing the two classes to be classified

We used an LSTM network whose input layer was designed to match the output of the CNN described before. Besides, the architecture of the LSTM was adopted to achieve a trade-off between performance and computational cost. Specifically, the network consisted of two recurrent layers, which means that it works like a stacked LSTM with the second receiving as input the outputs of the first LSTM and computing the final results [44]. Therefore, a hidden size of 20 was selected, which refers to the number of units in each LSTM cell. Afterwards, a dropout operation was applied to randomly discard 50% of the neurons during training. This prevents the overfitting of the network, forcing the model to diversify and not learn redundant information [45]. Finally, two fully-connected layers were used, the last one is the output layer with two neurons. Figure 4 depicts a schematic representation of the proposed LSTM network.

The performance of the proposed method is assessed in a five-fold stratified cross-validation scheme [46]. Thus, the data set is randomly split into 5 sets, each containing approximately the same percentage of CFS sequences from each class. In each iteration, four folds are aggregated to produce a training set, and the remnant fold is kept for evaluation. Four performance measures were obtained to evaluate the test fold in each case: specificity, sensitivity, balanced accuracy, and area under the ROC curve (AUC). These metrics use derivations from the confusion matrix such as true positive (TP), true negative (TN), false positive (FP), and false negative (FN). The specificity is computed as

15 $$\begin{aligned} S_{\text{1}} = \frac{n_\text{TN}}{n_\text{TN}-n_\text{FP}} \end{aligned}$$

and the sensitivity as

16 $$\begin{aligned} S_{\text{2}} = \frac{n_\text{TP}}{n_\text{TP}-n_\text{FN}} \end{aligned}$$

The imbalance of our data would lead to inaccurate outcomes if accuracy was used, so we opted for balanced accuracy which is resistant to class imbalance and is calculated as the mean of sensitivity and specificity. Finally, the AUC is employed to evaluate the ability of the model to identify the different classes.

#### Interpretability Method

**Fig. 5:**
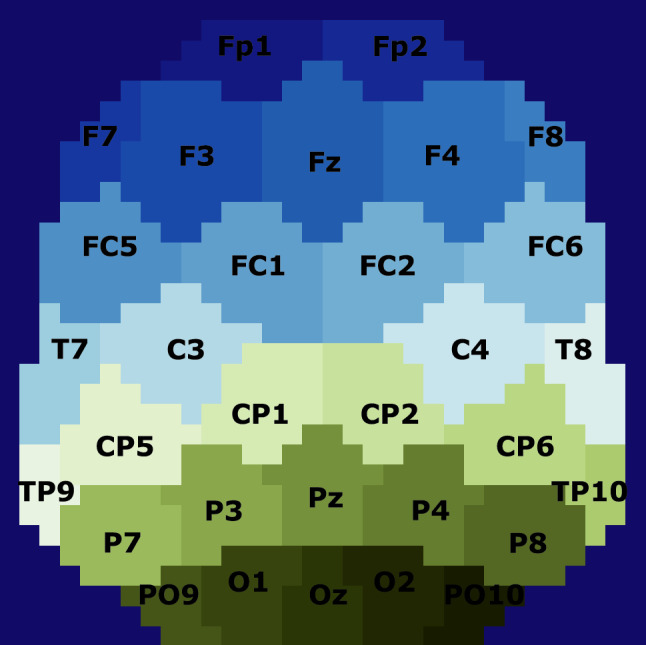
Feature mask to obtain the interpretable representations used in LIME. Each super–pixel contains a group of pixels related to the position of the corresponding EEG electrode

With these measures we assess the efficacy of the proposed model, however, understanding the reasons behind predictions is fundamental to improve the knowledge about the model. Therefore, we aim to achieve an explanation for the classification stage and provide information related to the data used. Consequently, this information will help to improve the understanding of the neural basis of DD thanks to the proposed methodology to transform EEG data into CFS images. We used local interpretable model-agnostic explanations (LIME) [47] to identify the most relevant regions during classification. LIME is model-agnostic and works by learning an interpretable model that is locally faithful to the classifier using a series of artificially generated data containing only a part of the original attributes. We have grouped pixels in a frame from the CFS sequences into super-pixels or segments, which correspond to interpretable features. The grouping is performed by considering in each feature segment the pixels most affected by the values of the corresponding electrode in the interpolation process used to create the CFS images. Figure 5 shows a feature mask with 31 super-pixels corresponding to the area of influence of each EEG electrode in the images. The feature mask is constructed following the same procedure described in Sect. 2.3 for a layer of a frame but putting a different constant value for each electrode. In this way, every pixel of a super-pixel has the same value indicating the feature group it belongs to.

The interpretable representation denoted as $x' \in \{0,1\}^{d'}$ used by LIME are binary vectors indicating if the segments in the instance being explained, $x \in \Re ^d$, are set to zero or maintain the original value. Thus, the explanation given by LIME is defined as

17 $$\begin{aligned} \xi (x) = {\mathop {{{\,\mathrm{arg\,min}\,}}}\limits _{g \in G}}\{{L(f,g,\pi _x) + {\varOmega} (g)}\} \end{aligned}$$

where the model being explained is *f*, *f*(*x*) the probability that *x* belongs to a certain class and $\pi _x$ a similarity or proximity measure. The interpretable model for the instance *x* is the model *g* from the class of potentially interpretable models *G* and $\varOmega (g)$ is a measure of its complexity. To train the interpretable model, LIME needs a set of perturbed samples *Z* obtained by drawing nonzero elements of the interpretable representation, $x'$, uniformly at random. Each perturbed sample $z'\in \{0,1\}^{d'}$ is transformed to the original representation $z \in \Re ^d$. The proximity between *z* and the instance being explained *x* is measured with $\pi _x$ and *f*(*z*) is obtained which is used as a label for the interpretable model. To ensure both interpretability and local fidelity, LIME minimises the fidelity function, $L(f,g,\pi _x)$, while having $\Omega (g)$ be low enough to be interpretable by humans. In this case, the locally weighted square loss is used as *L*, a linear Lasso model as an interpretable model, and an exponential kernel on top of the L2 or Euclidean distance for the proximity measure. The perturbed dataset contains $N=10,\!000$ samples, each $z'$ is the data to train the interpretable model that is weighted by the proximity measure and the output of our model *f*(*z*) is the target.

## Results

We have jointly employed CFS image sequence extraction and deep learning techniques to assess the differential characteristics of the neural processes involved in the experiment carried out. We have trained the proposed neural network architecture with image sequences from the EEG signals, that are obtained from measurements of CFS in each EEG channel along the temporal dimension. Our classification analysis reveals that the CFS image sequences created with phase synchronisation values from the combination of bands Theta-Gamma (TG), Alpha-Beta (AB), and Beta-Gamma (BG) achieve the best performance. Reaching up to $83\%$ balanced accuracy for the 4.8 Hz stimulus, followed by the 40 Hz stimulus with $72.4\%$ balanced accuracy. The performance achieved for each stimulus by the CFS image sequences and by its three layers separately estimated by balanced accuracy, specificity, sensitivity, and AUC is indicated in Table 2. These results are the average of the five fold from the stratified *K-*fold cross-validation scheme employed.

**Table 2:** Classification performance for the CFS sequences composed by layers of Theta-Gamma, Alpha-Beta, and Beta-Gamma CFS measures

CFS EEG bands	Stimulus	Balanced accuracy	Sensitivity	Specificity	AUC
TG-AB-BG	**4.8 Hz**	${\varvec{0.83\pm 0.077}}$	${\varvec{0.733\pm 0.17}}$	${\varvec{0.927\pm 0.064}}$	${\varvec{0.938\pm 0.03}}$
	16 Hz	$0.588\pm 0.086$	$0.4\pm 0.226$	$0.777\pm 0.117$	$0.732\pm 0.112$
	40 Hz	$0.724\pm 0.069$	$0.627\pm 0.155$	$0.822\pm 0.072$	$0.82\pm 0.07$
TG	4.8 Hz	$0.558\pm 0.095$	$0.433\pm 0.271$	$0.682\pm 0.351$	$0.609\pm 0.085$
	16 Hz	$0.534\pm 0.102$	$0.3\pm .194$	$0.767\pm 0.125$	$0.647\pm 0.148$
	40 Hz	$0.482\pm 0.029$	$0.173\pm 0.106$	$0.79\pm 0.133$	$0.527\pm 0.044$
AB	4.8 Hz	$0.718\pm 0.112$	$0.633\pm 0.163$	$0.803\pm 0.104$	$0.791\pm 0.112$
	16 Hz	$0.581\pm 0.029$	$0.367\pm 0.067$	$0.795\pm 0.027$	$0.608\pm 0.112$
	40 Hz	$0.753\pm 0.092$	$0.687\pm 0.168$	$0.819\pm 0.16$	$0.812\pm 0.095$
BG	4.8 Hz	$0.646\pm 0.084$	$0.533\pm 0.125$	$0.759\pm 0.134$	$0.693\pm 0.097$
	16 Hz	$0.722\pm 0.127$	$0.71\pm 0.146$	$0.765\pm 0.101$	$0.733\pm 0.226$
	40 Hz	$0.625\pm 0.117$	$0.533\pm 0.267$	$0.716\pm 0.155$	$0.696\pm 0.144$

Results are given for each of the three CFS layers separately and for the classification of the CFS sequence

The best result are in bold

**Fig. 6:**
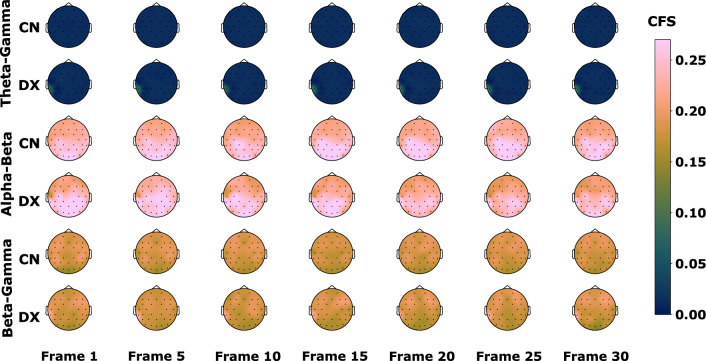
Average CFS topoplots in 4.8 Hz stimulus for control (CN) and dyslexic (DX) subjects measured between EEG bands Theta-Gamma, Alpha-Gamma and Beta-Gamma

These classification results arise from an exploratory analysis of the CFS intra-electrode. We explored the phase synchronisation between pairs of EEG frequency bands and its development through temporal segments. Figure 6 shows topoplots with the CFS measured for the best performing stimulus in classification: 4.8 Hz stimulus. These topoplots represent the average of each group phase synchronisation measured by Eq. (1) in each electrode for seven of the 30 temporal segments used in this work. Each pair of rows corresponds to topoplots for control and dyslexics for Theta-Gamma CFS first pair, Alpha-Beta CFS second pair, and Beta-Gamma CFS third pair. In other words, with these topoplots we can analyse the average CFS of each group, where a darker blue colour indicates lower CFS, as in the case of Theta-Gamma topoplots, and a pink colour a higher CFS, as in Alpha-beta topolots. In addition, we can see in each time segment how the CFS values change at each electrode. This is a step prior to the transformation into CFS image sequences.

**Fig. 7:**
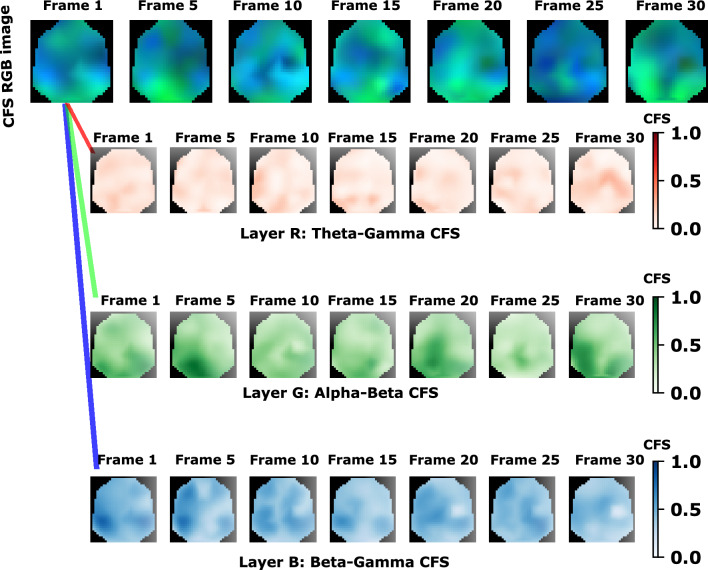
Example frames from the CFS image sequence for a control subject and its decomposition in the three CFS layers. Normalization is applied to aid visualisation

Then, the phase synchronisation measured at each electrode is employed to construct CFS images. As explained in Sect. 2.3, a layer contains the CFS measurements between two frequency bands at each electrode. By combining the layers from three frequency band pair CFS, we have created RGB images containing the joint phase synchronisation information at each electrode. Having segmented the EEG data, each RGB image constitutes a frame of the resulting image sequence. Figure 7 shows an example of seven frames from an image sequence of a control subject and its decomposition in the three CFS layers. The first layer frames are for Theta-Gamma, the second layer for Alpha-Beta, and the third for Beta-Gamma CFS. Each layer of the CFS images in this figure represents a plane with the projected EEG electrode locations. We then assigned the CFS value of each electrode to each point corresponding to its projected coordinates and interpolated the values in the convex hull defined by the projected electrode locations to generate the image. In this way, a darker color (red, green, or blue) in these layers means a higher CFS value, whereas a lighter colour a lower CFS. On the other hand, in the CFS RGB image, the CFS values of each layer are combined and function as an RGB image, i.e. it works as an additive model in which the colours are superimposed in space to reproduce a wide range of colours. When the CFS is zero for the same position in each layer it gives black, and the maximum CFS value produces white.

**Fig. 8:**
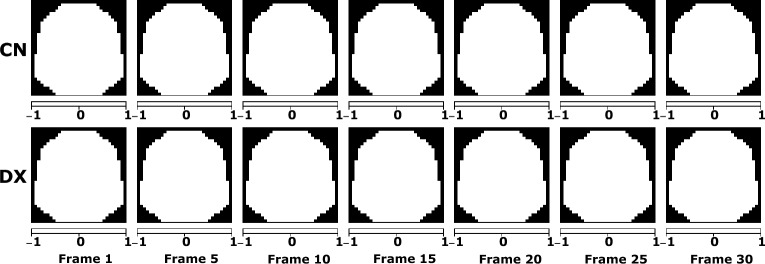
Five most important super-pixels in the average LIME maps obtained from the five fold used in cross-validation. The images used for averaging are those correctly classified in training and test sets

These CFS sequences have proven their differential capability at the classification stage (Table 2). Evidencing the existence of discriminating patterns in the intra-electrode phase synchronisation. We can use interpretability methods to identify the most relevant regions in the CFS sequences during classification. LIME is employed here to construct an explanation considering each frame of the sequence. Thus, for a sequence of images of a subject LIME is applied 30 times. In each iteration the perturbed samples are obtained by drawing different nonzero elements of one frame and keeping the remaining frames unchanged. With this approach, the temporal segments are considered in the explanation. Figure 8 shows a subset of seven frames from the sequence of images. Each frame represents the five most important super-pixels considering the correctly classified samples in each split of the stratified five-fold used in cross-validation. These super-pixels come from the feature mask shown in Fig. 5, where for each projected electrode location a cell including all nearest pixels is defined. The super-pixels depicted in Fig. 8 are those that contribute most positively (green) or negatively (red) to the classification of the given class. Hence, in each frame, the darker the colour (red or green) the more relevant that super-pixel is to the classification.

## Discussion

The transformation of EEG signals into images proposed in this work allows the application of advances in image classification methodologies. For this purpose, we have developed a two-stage deep learning architecture that exploits the information contained in the image sequences to extract spatial and phase synchronisation representations from each frame and temporal patterns from the sequence. In the first phase, for each CFS frame, we took advantage of the inherent characteristics of CNN to better utilise spatial information [39]. Then, by transforming the EEG signals into image sequences and using an LSTM network, we can obtain the temporal patterns of the sequence. The proposed architecture has been trained with image sequences derived from an exploratory CFS analysis of the EEG signal from low-level auditory processing of non-speech stimuli related to speech units such as syllables (4.8 Hz), intrasyllabic segmentation rhythms (16 Hz) and phonemes (40 Hz) of skilled and dyslexic seven-year-old readers. In the classification stage, the best results were obtained for the image sequences containing the CFS for Theta-Gamma, Alpha-Beta, and Beta-Gamma bands and the 4.8 Hz stimulus reached the best performance with an AUC of $93.8\%$ (Table 2).

This stimulus corresponds to the syllabic rate in Spanish language and engages the neural processes to transform continuous speech into a discrete code. This result is consistent with the temporal sampling hypothesis [6] where it is proposed an atypical processing of low-frequency modulations. Cognitive functions, such as listening to an auditory stimulus, engage neuronal oscillations simultaneously in multiple frequency bands that have distinct functional roles. The CFS is the mechanism used here to study the integration and coordination of information in neuronal processes distributed over frequencies [48] involved in auditory processing. CFS has been observed both locally within the sensor and globally between sensors located in different areas of the brain.

This CFS analysis exhibits differences in the average phase synchronisation between controls and dyslexics for the 4.8 Hz stimulus (Fig. 6). The CFS between Alpha-Beta bands has the highest values both for control and dyslexics of the three band pairs. The peak values are localised in the occipital and parietal lobes in both cases during the stimulus. Similar results have been found in CFS studies of EEG data during rest [49] and CSF during working memory tasks [10]. In dyslexics, there is a progressive decrease of phase synchronisation in left temporal and frontal lobes and a particular low CFS value for T7 electrode in the first temporal segments. For the Theta-Gamma bands, there is an increase of the phase synchronisation in electrode TP9 for dyslexic subjects that is maintained during the duration of the stimulus. This is also found for Alpha-Beta and Beta-Gamma CFS. Lastly, for Beta-Gamma CFS the highest average values move toward the frontal lobe in both hemispheres.

All the information from the CFS exploratory analysis is preserved in the transformation into images. It considers the locations of EEG electrodes based on the proposed method in [33] to preserve spatial information. With the novelty that phase synchronisation measures are considered to reveal patterns of coordination of neuronal oscillations in brain cognitive processes. Then, by creating an image sequence, the temporal evolution of these measures in each location over the duration of the stimuli is taken into account. Spectral information is also considered, as each frame of the CFS sequence is a three-channel image, and each layer corresponds to the CFS between different pairs of frequency bands. As this approach to obtain images from the EEG signals considers neural mechanisms, it is possible to extract information from the deep learning model developed and relate it to the neural processes involved. The interpretability and explainability of machine learning methods can facilitate a thorough understanding of the strategy for decision making and reason about the underlying learnt representations [50]. This is gaining relevance in the community of machine learning research, and it is highly demanded in the medical field [51, 52]. We adapted LIME for the explanation of sequences of images and proposed using a custom feature mask (Fig. 5) that relates the importance of each super-pixel to the location of the EEG electrodes. Thus, these super-pixels are linked to the phase synchronisation values in each electrode over time. This helps to locate the CFS patterns in the image sequences that contribute to the classification as control or dyslexic.

In this way, we can see in Fig. 8 that the FC5 electrode contributes the most to the classification as control in all frames. It is followed in importance by electrode CP1 which increases its contribution to the classification as control between frames 10 and 15. These two electrodes are located in the left hemisphere where there are key areas involved in normal reading [1, 53], suggesting that the CFS patterns in this hemisphere highly contributed for a subject to be considered in the control group. Electrodes TP9, CP2, Fp2, PO9, and P8 also have influence on the classification as control in certain frames. Contributing negatively to the classification as control, we encounter electrodes F3 and P4 in the first frame, F4 between the first and fifth frame, O1 in the last frame, FC2 between frames 20 and 25, and P3 from frame 10 onwards. In the case of dyslexia, electrode FC5 has an important negative contribution to the classification in all frames. Then, FC1, Pz, and PO9 spread their negative influence through the frames. All of them located in the left hemisphere. In contrast, we found super-pixels linked to electrodes located in areas of the right hemisphere that contribute to the classification as dyslexic such as electrodes F4, P4 until frame 20, and O1 between frames 20 and 25. Furthermore, electrode F3 in the left hemisphere contributes positively to the classification as dyslexic. This could relate the CFS patterns in these electrodes to recent evidence that language performance in dyslexics is compensated throughout neural mechanisms in the right hemisphere [53, 54]

Finally, some potential limitations of the present work can be discussed. First, the comparatively modest sample size of the dataset provided by the LEEDUCA research group should be taken into account. There are common difficulties in the recruitment of research subjects for studies with children with dyslexia, even when using EEG systems that facilitate working with children. In our case, we have achieved a sample size comparable to similar studies [55–57]. Secondly, the prevalence of DD induces a propensity to class imbalance that may lead to reduced sensitivity in classification. This is accounted for at the classification stage by employing a stratified *K*-fold cross-validation scheme. Lastly, the CFS images only represent the local phase synchronisation in each electrode. This gives information about the mechanism for linking activity that occurs at significantly different rates locally, but lacks the representation of connections between different brain regions.

## Conclusions and Future Work

The novel approach described in this work combines a measure of cross-frequency brain synchronisation dynamics, CFS, with a two-stage deep learning model for DD detection. Our main contribution is the transformation of EEG signals into image sequences considering neural mechanisms involved in low-level auditory processing. In particular, these images are obtained from a CFS analysis in intra-electrode brain rhythms derived from an EEG experiment with non-interactive auditory stimuli presented to skilled and dyslexic seven-year-old readers. Furthermore, we have designed a two-stage architecture that takes advantage of the spatial and temporal information contained in the image sequences to find discriminative patterns of phase synchronisation over time corresponding to these altered neural processes in low-level language processing in DD. Lastly, we have adapted the LIME interpretability method to the structure of the CFS image sequences using a novel feature mask to improve the understanding of the neural basis of DD by identify the most relevant regions during classification.

As a result, in the classification stage, the proposed two-stage model has reached up to 83% balanced accuracy for the 4.8 Hz stimulus, supporting the presence of discriminative patterns between control and dyslexic subjects. Consequently, linking the information from the CFS image sequences to the altered CFS patterns involved in low-level language processing that are present in the phonological deficit in DD. Additionally, we have obtained interpretable representations that allow us to identify the source (EEG electrode) and timing (frame) of these differential CFS patterns in auditory processing of typical and dyslexic readers. Revealing a link between CFS patterns with regions that are attributed to normal reading and those corresponding to compensatory mechanisms found in dyslexia. In this regard, we should restate the limitations of the present work: the reduced sample size of the dataset, although comparable to that of similar studies, as indicated in Sect. 4; the propensity to class imbalance due to the prevalence of DD; and CFS images only contain information about activity that occurs at significantly different rates locally. Nevertheless, these results pave the way to continue this line of research by planning future work such as exploring interelectrode CFS that would allow finding the interactions between the hemispheres, at the same time, the CFS measurement could be improved in spectral resolution by applying narrower bands in the filtering process. Finally, and most importantly, we intend to investigate a multimodal approach by combining information from different non-invasive neuroimaging techniques in the image sequences. This could be done with common low-cost techniques such as EEG and fNIRS.
